# Measuring the Usability of eHealth Solutions for Patients With Parkinson Disease: Observational Study

**DOI:** 10.2196/39954

**Published:** 2022-10-25

**Authors:** Jonas Bendig, Anja Spanz, Jana Leidig, Anika Frank, Marcus Stahr, Heinz Reichmann, Kai F Loewenbrück, Björn H Falkenburger

**Affiliations:** 1 Department of Neurology University Hospital and Faculty of Medicine Carl Gustav Carus Technische Universität Dresden Dresden Germany; 2 German Center for Neurodegenerative Diseases Dresden Germany

**Keywords:** eHealth, usability, Parkinson disease, telehealth and telemonitoring, older adults, aging, older population, neurodegenerative disease, digital solution, wearable sensor, mobile health, system usability, eHealth solution

## Abstract

**Background:**

Parkinson disease (PD) is a neurodegenerative disorder with a variety of motor and nonmotor symptoms. Many of these symptoms can be monitored by eHealth solutions, including smartphone apps, wearable sensors, and camera systems. The usability of such systems is a key factor in long-term use, but not much is known about the predictors of successful use and preferable methods to assess usability in patients with PD.

**Objective:**

This study tested methods to assess usability and determined prerequisites for successful use in patients with PD.

**Methods:**

We performed comprehensive usability assessments with 18 patients with PD using a mixed methods usability battery containing the System Usability Scale, a rater-based evaluation of device-specific tasks, and qualitative interviews. Each patient performed the usability battery with 2 of 3 randomly assigned devices: a tablet app, wearable sensors, and a camera system. The usability battery was administered at the beginning and at the end of a 4-day testing period. Between usability batteries, the systems were used by the patients during 3 sessions of motor assessments (wearable sensors and camera system) and at the movement disorder ward (tablet app).

**Results:**

In this study, the rater-based evaluation of tasks discriminated the best between the 3 eHealth solutions, whereas subjective modalities such as the System Usability Scale were not able to distinguish between the systems. Successful use was associated with different clinical characteristics for each system: eHealth literacy and cognitive function predicted successful use of the tablet app, and better motor function and lower age correlated with the independent use of the camera system. The successful use of the wearable sensors was independent of clinical characteristics. Unfortunately, patients who were not able to use the devices well provided few improvement suggestions in qualitative interviews.

**Conclusions:**

eHealth solutions should be developed with a specific set of patients in mind and subsequently tested in this cohort. For a complete picture, usability assessments should include a rater-based evaluation of task performance, and there is a need to develop strategies to circumvent the underrepresentation of poorly performing patients in qualitative usability research.

## Introduction

Parkinson disease (PD) is a neurodegenerative disorder characterized by a variety of motor and nonmotor symptoms. Despite the neurodegenerative nature of the disease, dopamine replacement therapy can drastically improve symptoms and quality of life, especially in the early stages of the disease [1]. With longer disease duration, symptoms often begin to fluctuate during the day, making the exact timing and dosage of medication more important [2]. eHealth solutions are becoming increasingly available, offering the potential to remind patients of their medications, assess the extent and timing of motor fluctuations, and ultimately help guide the decision for advanced therapeutic options such as deep brain stimulation and medication pumps [3-5]. In addition, eHealth solutions enable clinicians to assess patients over extended periods of time in their home environment. This method can help improve patient care but also provide more precise and more relevant end points for clinical trials [6].

A wide range of eHealth solutions has been examined in patients with PD, but most studies focus on selected subgroups of patients, such as those in earlier stages of the disease [7]. In the clinical routine, however, patients with PD are distributed across a wide range of age groups with diverse educational backgrounds, distinct motor impairments and—in many patients—important psychiatric and cognitive comorbidities [8]. There is a paucity of studies systematically investigating barriers for the successful implementation of eHealth solutions in the heterogeneous population of patients with PD [9].

In this context, usability research provides a variety of user-based methods that can be categorized into subjective and objective measures and quantitative and qualitative assessments [10]. Quantitative methods primarily include questionnaires and task completions, with questionnaires being the most frequently used method in eHealth research. The questionnaire with the broadest implementation among usability studies is the Systems Usability Scale (SUS) [11], which provides a subjective assessment of usability by the patient. Task completions provide objective information but require a system-specific setup, which can be difficult and time-consuming and potentially limits comparability. Qualitative methods include focus groups, interviews, and think-alouds. In contrast to quantitative methods, they can be more useful in identifying specific usability problems but suffer from a lack of comparability. Moreover, qualitative methods require trained evaluators and laborious data analysis. Think-alouds and qualitative interviews were the most frequently used methods in the usability testing of eHealth solutions [11]. For patients with PD specifically, usability assessments have mainly relied on questionnaires as well as adherence monitoring and mostly reported positive results for sensor systems and smartphone/tablet apps [12-15]. Although mixed methods approaches have become more common recently, there is substantial heterogeneity in the methods, and only a minority of studies focused specifically on the usability. In the broader context of chronic conditions, a recent systematic review concluded that the usability of wearable devices is poorly measured and reported [15]. Furthermore, there is no consensus regarding the methodology to assess usability in older adults, even though investigations about the sensitivity of different methods have been explicitly recommended [16].

Against this background, we aimed to identify which methods are suitable for comprehensive usability testing in our primarily older cohort of patients with PD and which factors can predict the successful use of devices for telemedicine and home monitoring. For this objective, we designed a mixed methods usability battery based on the most commonly used quantitative and qualitative methods for eHealth solutions and tested the usability of 3 different devices, including (1) a tablet app, (2) wearable sensors, and (3) a camera system.

## Methods

### Study Population

In all, 18 patients were recruited from the ward for movement disorders at the University Hospital Dresden between July 2020 and September 2021. Written informed consent was obtained from all participants before inclusion in the study. Inclusion criteria were the clinically probable diagnosis of idiopathic PD by a specialist for movement disorders according to the current guidelines of the International Movement Disorders Society [17] and sufficient German language skills. Exclusion criteria were the inability to walk and any psychiatric comorbidity that excluded the patients from participating in the study according to the discretion of the investigator.

### Ethics Approval

The study was approved by the institutional review board of Technische Universität Dresden, Germany (BO-EK-212052020).

### Tested Systems

We assessed 3 eHealth solutions that use different paradigms: (1) guided measurements at specific time points by a camera system; (2) continuous, implicit monitoring of mobility by wearable sensors; and (3) a combination of guided and continuous measurements by a tablet app. The systems were described in detail previously [18].

Briefly, the 3D-camera system (Motognosis Amsa; Motognosis GmbH) consisted of a stand-alone PC and a depth camera (Microsoft Kinect; version 2). Patients were instructed to perform motor exercises by prerecorded videos and audio instructions. Kinematic parameters were derived from the exercises to describe patients’ mobility and symptoms.

The wearable system (PD Neurotechnology Ltd) consisted of five 9-axis inertial measurement unit sensors, worn on wrists, shanks, and the trunk. The data from the sensors were used to analyze patients’ motor status [19]. The PDMonitor mobile app was not used in this study nor was the device used for motor symptom clinical assessment and treatment modification.

The tablet app (TelePark tablet app; Intecsoft group) included a medication alert, questionnaires, fall documentation, activity documentation, and a task reminder.

The tablet app represented a system that was still at an early stage of development, whereas the 3D-camera system and the wearable sensors were already fully developed and licensed medical products.

### Study Schedule

**Figure 1:**
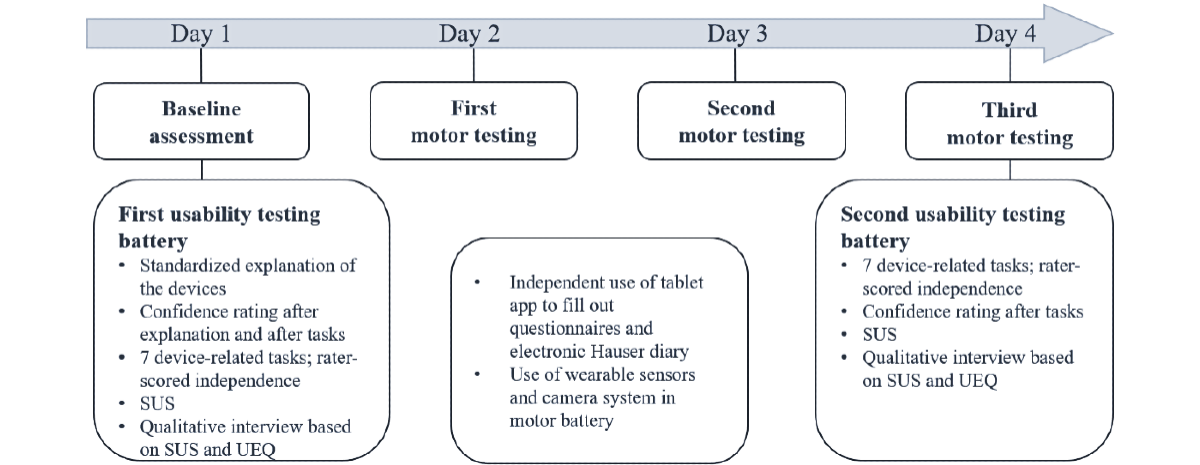
Schematic overview of the study schedule. To keep the assessments efficient, only 2 of the 3 devices (tablet app, camera system, and wearable sensors) were tested per patient, resulting in 3 groups of patients that used the same set of devices. UEQ: User Experience Questionnaire; SUS: System Usability Scale.

Inpatients completed 4 assessments on 4 days within a maximum period of 7 days during their stay at the movement disorders ward (Figure 1). On day 1, patients performed the baseline assessment and the first mixed methods usability testing battery (detailed below). Routine motor testing was carried out on days 2 and 3 (detailed below). Patients were filmed with the camera system and wore the wearable sensors during the motor testing sessions. Between assessments, patients used the TelePark app to complete questionnaires and an electronic version of the Hauser diary [20]. The wearable sensors and the camera system were only used or worn during the motor tests and usability batteries. Patients were encouraged to put on or remove the sensors independently but received help from the study personnel if requested. On day 4, patients performed a final round of motor testing and the second usability testing battery.

### Baseline Assessment

Patients were assessed with rater-based scales and self-report questionnaires to evaluate motor and cognitive function as well as eHealth literacy. The questionnaires were filled out digitally by the patients in the TelePark app. If patients were not able to independently complete the questionnaires on the tablet, they were supported by the investigators. The following scales and questionnaires were used in the baseline assessment: the Freezing of Gait Questionnaire (FOG-Q) [21], Hoehn and Yahr scale [22], Unified Parkinson’s Disease Rating Scale III (UPDRS III) [23], Beck Depression Inventory-II [24], Montreal Cognitive Assessment (MOCA) [25], and eHealth Literacy Scale (EHEALS) [26]. The total score of the EHEALS ranges between 8 and 40, with higher scores indicating higher self-perceived eHealth literacy.

### Motor Testing

Motor testing consisted of a UPDRS III, a timed up-and-go test [27], a freezing of gait test [28], a Mini-BESTest [29], fast 360° turns, the video-instructed Motognosis Amsa protocol (finger tapping, stand up and sit down, stance with closed feet, comfortable 360° turns, stepping in place, short comfortable speed walk, and short maximum speed walk), and the operator-instructed Motognosis PASS-PD protocol (finger tapping, hand grasping, arm holding, finger-nose test, foot tapping, stand up and sit down, stance with closed feet, comfortable 360° turns, stepping in place, comfortable walk, and maximum speed walk). During the period of the assessment, patients were filmed by the 3D-camera system and wore the wearable sensors.

### Usability Testing Battery

The usability testing battery was performed on the first and the last day of the study. To reduce patient burden, each patient assessed the usability of only 2 of the 3 study components (tablet app and camera, tablet app and wearables, or camera and wearables). The devices were assigned randomly to the patients by a prespecified permuted list. Usability was assessed for each device separately.

First, patients were given a standardized explanation of the device. Patients were then instructed to carry out 7 device-related tasks, which covered all important functions of the systems, as independently as possible. These tasks were setting up the camera and performing different tasks in the Amsa protocol (camera system), putting on the sensors and handling the charging procedure and the data transfer processes (wearable sensors), and using all relevant functions in the app (tablet app). The execution of the tasks was observed by the investigators and rated on a 6-item ordinal scale according to the independence of task execution (ranging from 5=“Does not need help; does not consult manual” to 0=“Can contribute nothing or almost nothing to the implementation of the task”). The sum of all 7 independence ratings was transformed into a rater-based independence score ranging from 0% (no independent use in any tasks) to 100% (fully independent use in all tasks) with the following formula:

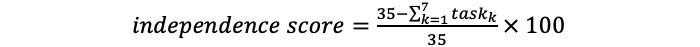



After the task-related device testing, patients filled out the SUS [30] and were asked again how confident they felt now to use the devices alone in a home monitoring setting (confidence score from 0% to 100%). The SUS is a 10-item Likert scale to assess subjective usability, containing questions such as the perceived complexity of a system, the user’s confidence in using a system, or its learnability. The SUS has been widely used, and normative data exist allowing SUS ratings to be positioned relative to other systems [31]. Furthermore, it has been shown that the SUS can provide valid scores even with small sample sizes [32].

Finally, we conducted an interview based on domains of established usability instruments (SUS and user experience questionnaire [33]). This interview took 15 to 30 minutes and consisted of 12 open-ended questions concerning the following domains: attractivity, independent use, learnability, perspicuity, efficiency, stimulation, and novelty. For each domain, the patients were asked 2 open-ended questions about their opinion on the domain quality and about improvement suggestions in that specific domain. The same procedure was then carried out for the second device.

### Data Analyses and Sample Size

Data are depicted as median with 25th and 75th percentile or as mean with SD depending on data normality as assessed by a visual inspection of histograms. To assess the differences between the systems, a Kruskal-Wallis test with post hoc Dunn test was used. Due to the small sample size and the exploratory nature of the study, no correction for multiple testing was used. Predictors of successful use were identified by correlation analysis (Spearman *ρ*). Significant correlations (*P*<.05) were visualized in a network graph with the *ForceAtlas2* algorithm [34]. The temporal stability of usability outcomes was assessed by comparing the first and the second measurement with a Wilcoxon signed-rank test. Data visualization and statistical analyses were performed with Python (*Statsmodels*, *Scipy*, *Matplotlib*, and *Seaborn* packages) and Gephi software. The sample size of 12 patients per system was determined using guidelines for conducting qualitative research [35].

## Results

In total, 19 patients were included in the study, and 1 patient dropped out after the first usability battery due to personal reasons (not named). The clinical and demographic data of the remaining 18 patients are summarized in Table 1.

**Table 1:** Clinical and demographic data. Data are presented as mean with SD or median with absolute range.

		Value
Patient, n		18
Age (years), median (range)		69 (37-86)
**Sex (N=18), n (%)**		
	Female	7 (39)
	Male	11 (61)
Hoehn and Yahr stage, median (range)		3 (1-4)
Disease duration (years), mean (SD)		11 (7.3)
UPDRS III^a^ score, mean (SD)		27 (9.0)
MOCA^b^ score, mean (SD)		25 (2.7)
EHEALS^c^ score, mean (SD)		23 (8.8)
BDI-II^d^ score, mean (SD)		12 (7.4)
FOG-Q^e^ score, mean (SD)		11 (5.1)

^a^UPDRS III: Unified Parkinson’s Disease Rating Scale III.

^b^MOCA: Montreal Cognitive Assessment.

^c^EHEALS: eHealth Literacy Scale.

^d^BDI-II: Beck Depression Inventory-II.

^e^FOG-Q: Freezing of Gait Questionnaire.

### Testing Usability Measures

**Figure 2:**
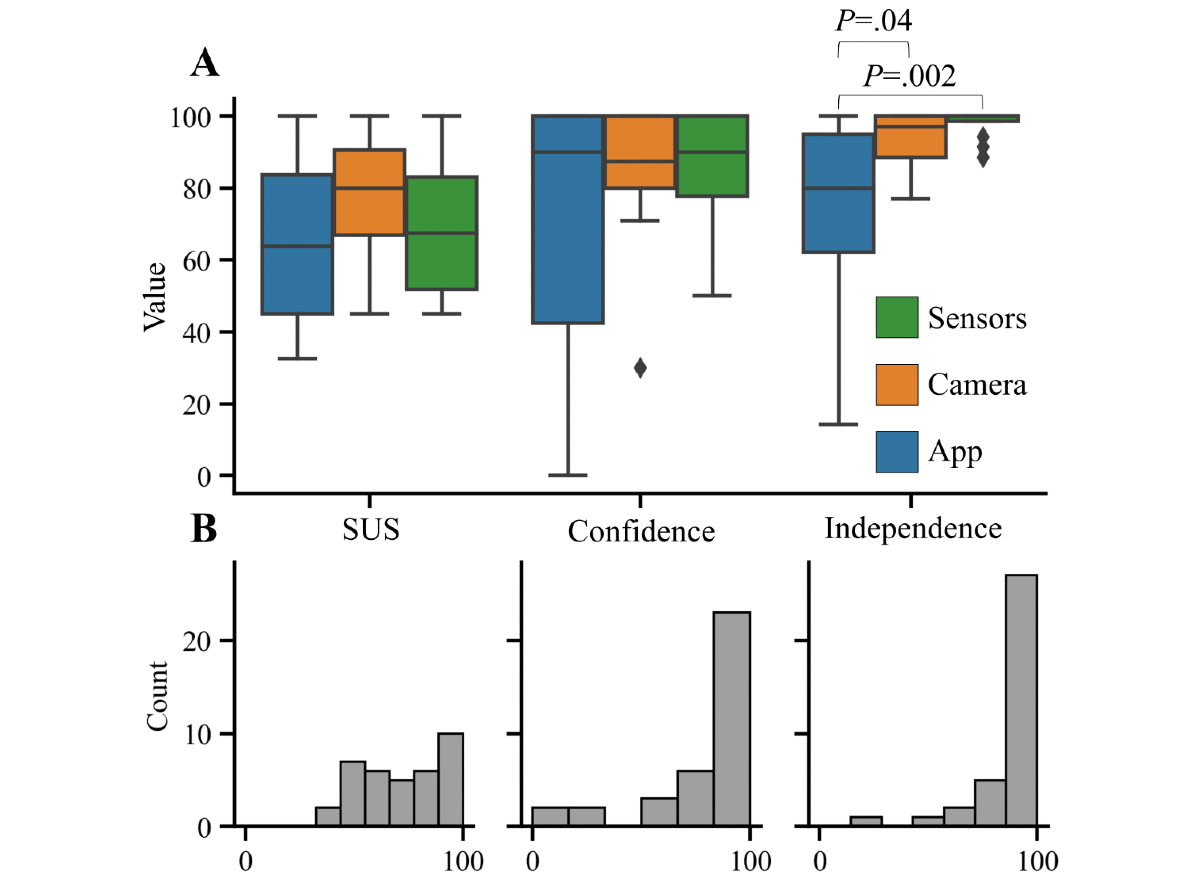
Comparison of independence scores, confidence scores, and SUS scores from the second usability battery: (A) Box plots and (B) histograms. *P* values were from Dunn test without correction after significant Kruskal-Wallis test. Box plots depict median (black line), IQR (boxes), range (whiskers), and outliers (diamonds; >75th percentile + 1.5 IQR or <25th percentile – 1.5 IQR). SUS: System Usability Scale.

The SUS is a widely used score for a quick and simple assessment of usability [31]. SUS scores (second usability battery) did not differ significantly between devices (*P*=.34, Kruskal-Wallis test; Figure 2A). In addition, we compared the empirical confidence scores (patient-rated) and the task-based independence scores (investigator-rated) between the 3 systems (Figure 2A). The confidence scores and the independence scores showed a pronounced ceiling effect, whereas the SUS scores were more evenly distributed (Figure 2B). Exclusively, the independence scores differentiated between the app (ie, the device that is still at an early stage of development) and the fully developed and licensed systems (*P*=.006, Kruskal-Wallis test; post hoc tests in Figure 2A). For the subsequent correlation analyses in this study, we therefore selected the objective independence score as the most relevant measure of successful use.

### Identifying Predictors for Successful Use

**Figure 3:**
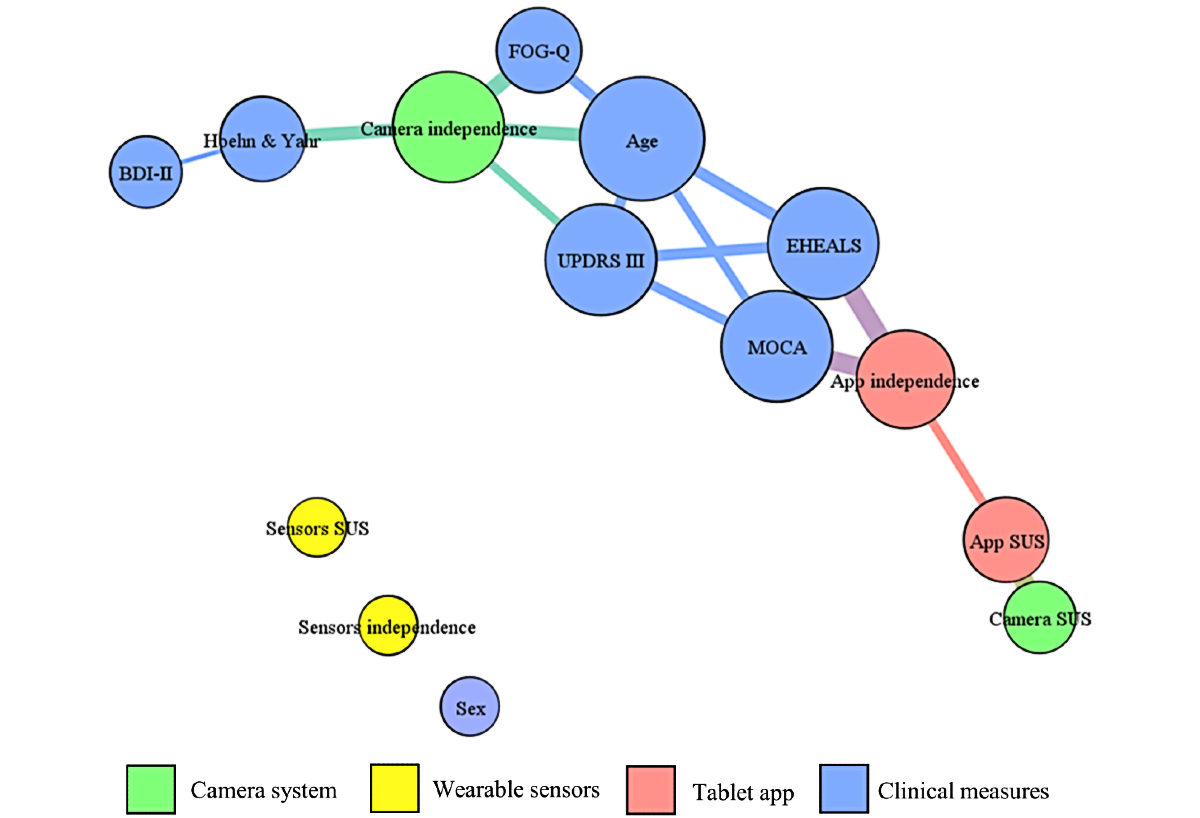
Network graph of correlations (Spearman *ρ*) between baseline variables, SUS scores, and independence scores from the second usability battery. Only significant correlations (*P*<.05, uncorrected values) are included the network. The relative size of the variables indicates the absolute number of connections. The thickness of the connections indicates the magnitude of the correlation (thicker lines indicate stronger correlations). BDI-II: Beck Depression Inventory; EHEALS: eHealth Literacy Scale; FOG-Q: Freezing of Gait Questionnaire; MOCA: Montreal Cognitive Assessment; UPDRS III: Unified Parkinson's Disease Rating Scale III; SUS: System Usability Scale.

To identify factors that predict whether patients are able to use a device well, we plotted correlation matrices to explore the interdependence between the rater-based independence score, the SUS, and baseline parameters. In addition to the independence scores and SUS scores, the following variables were used in the correlation analysis: age, sex, Hoehn and Yahr stage, UPDRS III, FOG-Q, MOCA, and EHEALS. The network graph of correlations visualizes that the rater-based independence scores for wearable sensors (yellow), camera system (green), and tablet app (red) do not cluster together (Figure 3). This visualization indicates that the prerequisites for successful use differ between the 3 systems. The independent use of the wearable sensors did not correlate significantly with any clinical characteristics (age: *P*=.07; sex: *P*=.38; Hoehn and Yahr stage: *P*=.44; UPDRS III: *P*=.59; FOG-Q: *P*=.94; MOCA: *P*=.40; EHEALS: *P*=.68), but only 3 (25%) out of 12 patients were not able to use the system fully independently. This finding implies that the sensors were usable for most of the patients regardless of their clinical characteristics. The independent use of the camera system correlated strongly with age and motor scores (FOG-Q, UPDRS III, and Hoehn and Yahr stage), and the independent use of the tablet app showed strong correlations with cognition (MOCA) and eHealth literacy (EHEALS). Table 2 shows the strongest correlations with the rater-based independence score for each system.

In contrast to the rater-based independence scores, we found no significant correlations between the subjective and more variable SUS scores with the clinical measures for the tablet app (age: *P*=.79; sex: *P*=.89; Hoehn and Yahr stage: *P*=.85; UPDRS III: *P*=.92; FOG-Q: *P*=.14; MOCA: *P*=.28; EHEALS: *P*=.07), the wearable sensors (age: *P*=.78; sex: *P*=.15; Hoehn and Yahr stage: *P*=.52; UPDRS III: *P*=.99; FOG-Q: *P*=.12; MOCA: *P*=.96; EHEALS: *P*=.19), or the camera system (age: *P*=.45; sex: *P*=.70; Hoehn and Yahr stage: *P*=.62; UPDRS III: *P*=.16; FOG-Q: *P*=.49; MOCA: *P*=.99; EHEALS: *P*=.26).

**Table 2:** Correlations of clinical characteristics with independent use. The 3 strongest correlations (Spearman *ρ*) with *P* values between clinical characteristics and independence scores for the 3 systems are shown.

Device, clinical characteristic		Spearman *ρ*	*P* value
**Tablet app**			
	EHEALS^a^	0.90	<.001
	MOCA^b^	0.89	<.001
	Age	–0.63	.03
**Camera system**			
	FOG-Q^c^	–0.80	.002
	Hoehn and Yahr	–0.72	.009
	Age	–0.71	.009
**Wearable sensors**			
	—^d^	—	—

^a^EHEALS: eHealth Literacy Scale.

^b^MOCA: Montreal Cognitive Assessment.

^c^FOG-Q: Freezing of Gait Questionnaire.

^d^For the wearable sensors, no significant correlations were found.

### Temporal Change in Usability Outcomes

To assess the system-specific learnability and stability of the usability outcomes, we compared usability outcomes between the first and second round of the usability battery on days 1 and 4, respectively. For the tablet app, independence and confidence scores did not differ significantly between the 2 time points (independence: mean 79.5%, SD 25.6% vs 75.5%, SD 25.8; *P*=.34; confidence: mean 75.4%, SD 24.3% vs 69.6%, SD 38.8%; *P*=.29). The camera system, in contrast, had a significantly higher confidence score in the second usability battery (mean 63.3%, SD 32.5% vs 84.7%, SD 19.8 %; *P*=.008); independence scores were high at both time points (mean 89.3%, SD 14.8 % vs 93.8%, SD 7.9%; *P*=.12). The wearable sensors showed a significantly higher independence score in the second usability battery (mean 91.4%, SD 9.2% vs 97.9%, SD 4.1%; *P*=.03); confidence ratings did not change (mean 79.5%, SD 25.6% vs 75.5%, SD 25.8%; *P*=.67). SUS scores did not change significantly between the 2 time points for any of the tested systems (tablet app: *P*=.18; camera system: *P*=.20; wearable sensors: *P*=.88). The system-specific changes in usability outcomes indicate a different learnability for each individual system and underscore the importance of longitudinal usability assessments. Furthermore, they suggest that performance and confidence may differ. The lack of difference in the SUS scores between the 2 time points is consistent with the lack of difference in the SUS scores between the 3 systems (Figure 2), suggesting that the SUS can miss important aspects of usability.

### Influences on Qualitative Interviews

**Figure 4:**
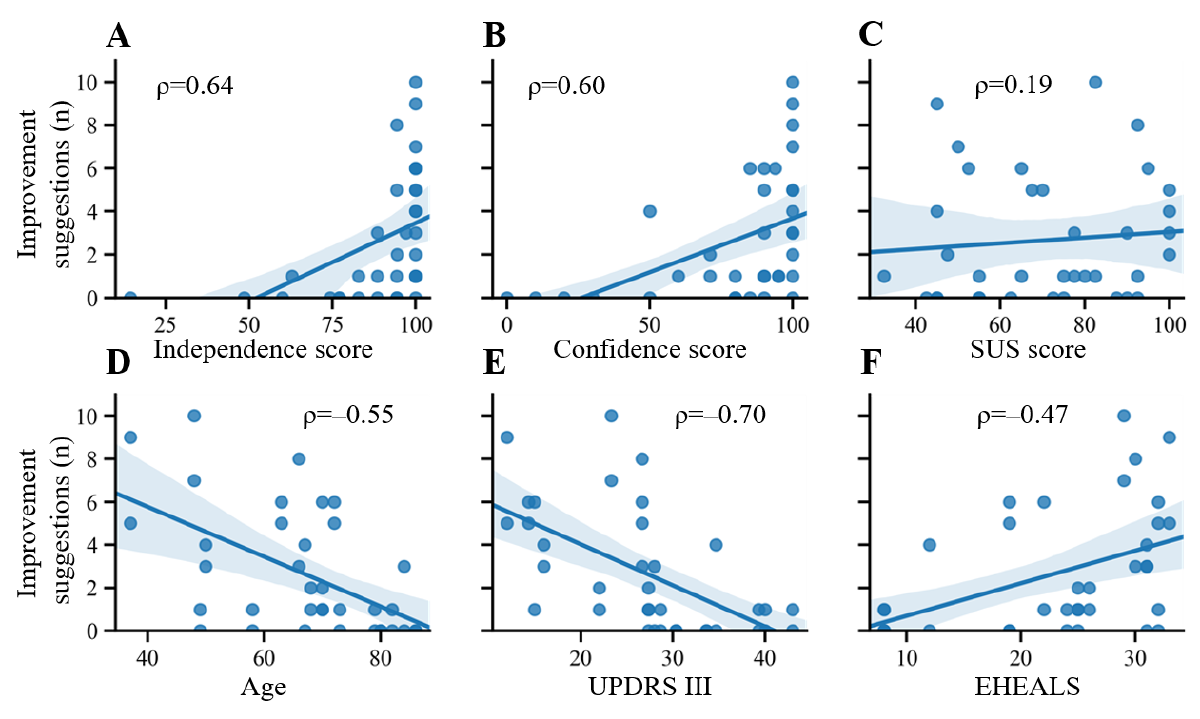
Linear regression plots of valid improvement suggestions with (A) independence scores, (B) confidence scores, (C) SUS scores, and (D-F) clinical characteristics. The strength of the correlation (Spearman *ρ*) is indicated in the plot. All *P* values for the Spearman correlations were <.001 except for the SUS score (*P*=.11). Individual values for each system are plotted. Improvement suggestions are aggregated from both usability measurements for each system. EHEALS: eHealth Literacy Scale; UPDRS III: Unified Parkinson's Disease Rating Scale III; SUS: System Usability Scale.

In the qualitative section of the first and second usability batteries, patients were asked about improvement suggestions for the eHealth solutions. To determine predictors of qualitative feedback, we counted the total number of unique improvement suggestions per patient and correlated them with usability outcomes and clinical characteristics. We found moderate-to-strong and highly significant correlations with independence scores, confidence scores, eHealth literacy, motor phenotype, and age (Figure 4). These correlations suggest that patients who were able to use the devices well gave more valid improvement suggestions than patients who did not. Patients giving more feedback were also younger, had lower motor disability, and higher eHealth literacy. SUS scores did not correlate with the number of improvement suggestions, suggesting that the subjective rating of an eHealth solution does not affect the number of improvement suggestions.

## Discussion

### Principal Findings

In this study, we performed a comprehensive usability battery on 3 eHealth solutions, using subjective and objective assessments. The objective rater-based evaluation of tasks (independence score) discriminated better between the different eHealth solutions than the subjective quantitative usability scale (SUS). Moreover, the successful use of each eHealth solution was associated with specific clinical characteristics—notably, cognitive ability and eHealth literacy for the tablet app or motor ability and age for the camera system. Finally, most improvement suggestions were provided by patients who were able to use the eHealth solutions well.

### Comparison Between Usability Measures

There is a paucity of data on the sensitivity of usability testing methods [16], and optimal methods for specific eHealth solutions or cohorts have not been identified [11]. We therefore compared usability as reported by the quantitative and easy-to-use SUS with patient-rated confidence and investigator-rated independence in prespecified settings. Given that the 3 eHealth solutions investigated here (tablet app, camera system, and wearable sensors) differed strongly in complexity and development stage, we expected to find differences in all methods. However, only the rater-based independence scores showed a significant difference between the 3 technologies. The ceiling effect of the independence scores could indicate that the systems were indeed easy to use for many patients. Alternatively, the prespecified tasks were not hard enough. As the tasks were developed to cover all relevant functions of each system, we interpreted this ceiling effect as successful use. The SUS score did not show a ceiling effect, but it did not differentiate between the fully developed systems and the less developed system in our study. Furthermore, the SUS did not reflect the increased confidence and independence between the first and second time point of testing. Collectively, these findings are in line with similar studies, where successful use was not associated with higher SUS scores [36,37]. These findings suggest that this well-established scale could potentially miss important information in the population of patients with PD, and in other populations of older and cognitively impaired people. The recent development of a simplified SUS score for older adults is in line with this interpretation [38]. 

The improvements in confidence or independence scores for the camera system and the wearable sensors indicate that even in a short period of 4 days, older adults (1) are able to change their perspective toward eHealth solutions and (2) can learn to handle such systems. The lack of improvement for the tablet app shows that learnability is dependent on the eHealth solution, which is in line with previous results from other studies [39]. These results should caution researchers to not rely on a single test to predict successful use. Based on our results, we recommend a short rater-based test, a subjective patient-rating validated in older adults (eg, a questionnaire), and a trial period for each patient and device before applying eHealth solutions in trials or clinical practice.

### Predictors of Successful Use

Predictors of successful use differ strongly between individual eHealth solutions (Figure 3). For the app, the strong associations with cognitive function and eHealth literacy indicate that both constructs need to be considered in the design of such mobile health systems with a largely software-based interface [40,41]. Hence, eHealth solutions should be developed with a specific range of cognitive function and eHealth literacy in mind and then should be tested and marketed for this group of patients. For the camera system, older patients with more severe motor symptoms had more problems, whereas the wearable sensors were usable for most patients independent of clinical characteristics. This finding is not surprising given the mainly physical nature of interacting with the wearable sensors or performing guided tasks in front of the camera. In contrast, sensors were usable by most patients regardless of their clinical characteristics. Collectively, our findings align well with the MOLD-US framework, where usability prerequisites for the app system fall into the domains of cognition and motivation and prerequisites for the camera system are associated with the physical ability [42].

The subjective aspect reported by the SUS is necessary for a patient to start the use of eHealth solutions to avoid attrition with continual use [10], and indeed, SUS scores varied considerably between participants (Figure 2). We sought to determine predictors of SUS scores. However, we were not able to determine predictors for subjective usability scores as reported by SUS, likely due to the small sample size (n=12 per system) in our study. We only observed for the app an association between the SUS and independence scores in the graph analysis (Figure 3). This analysis, therefore, needs to be addressed in subsequent studies with more participants. Moreover, attrition could not be assessed in this short and highly standardized paradigm.

### Improvement Suggestions

We found a strong positive correlation between successful use and a patient’s ability to advise on possible improvements of the tested systems during the qualitative interview (Figure 4A). In other words, suggestions came mainly from individuals that did not have problems using the system. Therefore, established methods such as “think-alouds” or “focus groups” could suffer from an overrepresentation of opinions voiced by well-performing, mildly affected patients. It is not clear whether following these suggestions will improve or worsen usability for those who have trouble using the system successfully. The method of counting the total number of improvement suggestions does not take into account the quality of the suggestions; thus, the presented results should be reinvestigated more thoroughly in future studies. Furthermore, the reported correlation could also be mediated or moderated by the factors age, disease severity, cognitive status, or eHealth literacy (Figure 4D to F). However, inferring causal connections between highly interconnected variables was beyond the scope of this study, and to our knowledge, there are currently no articles that have comprehensively assessed the effects of these variables in the context of qualitative usability research. With an aging population in Western countries and a predicted rise in patients living with neurodegenerative diseases [43,44], a critical assessment of qualitative usability methods in the context of the target group is warranted.

### Limitations

Limitations of our study include the small sample size, only a single recruitment site, and the controlled inpatient setting. Furthermore, the comparison of different methods is based on a subset of the existing tools that does not include techniques such as think-alouds, focus groups, or alternative measures of efficiency (eg, time to complete a task). This limitation reduces the generalizability of our findings and warrants further investigation with different systems, settings, and patient cohorts. Moreover, the high correlations between eHealth literacy, motor symptoms, cognitive impairment, and age limit the causal interpretability of the obtained results.

### Conclusions

The successful use of eHealth solutions in patients with PD is highly dependent on system-specific and patient-specific characteristics. Considering the growing field of digital health and the already existing abundance of different solutions for patients with PD [4,45,46], researchers and industrial partners need to consider the heterogeneity of patients and design eHealth solution for a specific constellation of age, cognitive and motor function, as well as eHealth literacy, and these criteria can be helpful for physicians in selecting the best solution for each individual patient.

## Abbreviations

EHEALS: eHealth Literacy Scale
FOG-Q: Freezing of Gait Questionnaire
MOCA: Montreal Cognitive Assessment
PD: Parkinson Disease
UPDRS III: Unified Parkinson’s Disease Rating Scale III
SUS: System Usability Scale
